# Therapeutic potential of HERS spheroids in tooth regeneration

**DOI:** 10.7150/thno.44782

**Published:** 2020-06-12

**Authors:** Yufeng Duan, Xuebing Li, Sicheng Zhang, Shikai Wang, Tao Wang, Hong Chen, Yan Yang, Sixun Jia, Guoqing Chen, Weidong Tian

**Affiliations:** 1 State Key Laboratory of Oral Disease, West China Hospital of Stomatology, Sichuan University, Chengdu, China.; 2 National Engineering Laboratory for Oral Regenerative Medicine, West China Hospital of Stomatology, Sichuan University, Chengdu, China.; 3 National Clinical Research Center for Oral Diseases, West China Hospital of Stomatology, Sichuan University, Chengdu, China.; 4 Department of Oral and Maxillofacial Surgery, West China Hospital of Stomatology, Sichuan University, Chengdu, China.; 5 School of Medicine, University of Electronic Science and Technology of China, Chengdu, China.

**Keywords:** Hertwig's epithelial root sheath (HERS), 3D culture, spheroid, HIF-1 pathway, tissue regeneration

## Abstract

Hertwig's epithelial root sheath (HERS) plays indispensable roles in tooth root development, including controlling the shape and number of roots, dentin formation, and helping generate the cementum. Based on these characteristics, HERS cell is a potential seed cell type for tooth-related tissue regeneration. However, the application is severely limited by a lack of appropriate culture methods and small cell numbers.

**Methods:** Here, we constructed a 3D culture method to expand functional HERS cells into spheroids, and investigated characteristics and application of dental tissue regeneration of these spheroids. HERS spheroids and HERS cells (2D monolayer culture) were compared in terms of biological characteristics (such as proliferation, self-renewal capacity, and stemness) *in vitro* and functions (including differentiation potential and inductive ability of dentin formation) both* in vitro* and *in vivo*. Further, transcriptome analysis was utilized to reveal the molecular mechanisms of their obvious differences.

**Results:** HERS spheroids showed obvious superiority in biological characteristics and functions compared to 2D monolayers of HERS cells *in vitro*. *In vivo*, HERS spheroids generated more mineralized tissue; when combined with dental papilla cells (DPCs), HERS spheroids contributed to dentin-like tissue formation. Moreover, the generation and expansion of HERS spheroids rely to some degree on the HIF-1 pathway.

**Conclusion:** HERS spheroid generation is beneficial for functional HERS cell expansion and can provide a useful cell source for further tooth regeneration and mechanistic research. Notably, HIF-1 pathway plays a critical role in HERS spheroid formation and function.

## Introduction

Hertwig's epithelial root sheath (HERS), a fleeting and transient double-layered epithelial structure that originates mainly from the outer and inner enamel epithelium of the enamel organ, plays an indispensable role in the formation of tooth root and periodontium 1, 2. Importantly, tooth root and periodontium tissues anchor teeth in the alveolar bone, which is the basis of functional mastication and pronunciation 2, 3. However, due to the high prevalence of periodontitis and the inability of existing treatments to fully reverse the disease or restore functional teeth completely, many people suffer from periodontitis and even tooth loss 4. Cell-based tissue regeneration has been considered a promising solution 4-6. HERS cells play an indispensable role in tooth root development, controlling the shape and number of roots and assisting in cementum generation 2, 7. HERS cells are also important candidate seed cells for dental tissue regeneration. However, unlike mesenchymal stem cell use, HERS cell application is limited by small cell numbers and lack of suitable culture methods 5, 6.

Previously, we achieved expansion of HERS cells on polystyrene dishes, but this method still requires optimization 8. For instance, the relatively long period of trypsin digestion required for cell passage can damage the cell membrane, and expanding the cells for more than three passages is difficult. A more efficient expansion method for HERS cells is therefore essential for further research and application. Considering the advantages of 3D cultured stem cells, including enhanced cell-cell interactions, well-maintained innate characteristics, up-regulated stem-cell-related genes, and stimulated cell proliferation 9-13, we propose that 3D culture of HERS cells may be a better method. In this study, we constructed the HERS spheroids culture methods (HSCM) and investigated cell characteristics and regeneration potential of these spheroids.

## Materials and methods

All experiments were approved by the Ethics Committee of West China Hospital of Stomatology, Sichuan University.

### Cell isolation, culture and harvest

Primary HERS cells and DPCs in the 2D monolayer culture environment were obtained and cultured as previously described 14. HERS primary cells at passage 1 (P1) were divided into two parts and further cultured using HSCM or 2D methods. DPCs at passage 5 (P5) were used for experiments.

For HERS spheroids culture, on day 1, after cells were mixed thoroughly on ice with an equal volume of Matrigel^TM^ Basement Membrane Matrix (referred to as Matrigel) (BD Biosciences, USA), 100 μl mixed Matrigel (2×10^5^ cells/ml) was dropped in the center of each well in 12-well plates and incubated at 37 °C for 30 min. Then, 1 ml epithelial cell medium (ScienCell, CA, USA) was supplemented into each well and refreshed every two days. The diameter of the spheroids was measured using an Olympus microscope IX71 with CellSens Standard Software (Olympus, Japan). HERS spheroids were harvested through two steps. First, most of the spheroids were released from the Matrigel by pipeting up and down, after which they were collected by centrifugation. Then, HERS spheroids were incubated with 0.25% trypsin for 3 min to further release them from the Matrigel.

2D monolayer HERS cells or HERS spheroids from day 1 were used for treatment with Dimethyloxallyl Glycine (DMOG) (MCE, USA) and BAY-87-2243 (MCE, USA) at a concentration of 100μM/ml and 5 μM/ml for 7 days respectively.

### Cell proliferation assay

The proliferation capacity of HERS cells and digested HERS spheroids was measured by Cell Counting Kit-8 (Dojindo, Japan) according to the manufacturer's protocol. Cells were seeded in 96-well plates at a density of 5×10^3^ cells/well. After 2 h of incubation with CCK8 at 37 °C, absorbance at 450 nm was detected with a spectrophotometer (Thermo Fisher Scientific Inc, USA) at the indicated time points. All tests were performed in triplicate, at minimum.

### Colony-forming unit assay

The colony-forming unit (CFU) assay was conducted to measure the self-renewal ability of 2D monolayer HERS cells and HERS spheroids. On day 1, 1×10^2^ cultured HERS cells from the two different culture methods were seeded into 60 mm dishes. On day 10, cells were fixed with 4% paraformaldehyde and then stained with crystal violet. Colonies containing 20 or more cells were scored. All tests were performed in triplicate, at minimum.

### TUNEL assays

TdT-UTP nick end labeling (TUNEL) assays were performed to detect apoptosis of target cells with the One Step TUNEL Apoptosis Assay Kit (Beyotime, China) according to the manufacturer's instructions. Briefly, cells were fixed with 4% paraformaldehyde for 30 min and then treated with 0.3% Triton X-100 for 5 min at room temperature, followed by TUNEL for 1 h at 37 ℃, protected from light. Next, DAPI was used to label the nuclei, and the FITC-labeled TUNEL-positive cells were imaged and counted.

### Mineralization induction

2D monolayer HERS cells and HERS spheroids were seeded into 6-well plates with 3×10^5^ cells per well. When cells reached 60-70% confluence, the culture medium was switched to induction medium (complete epithelial cell medium supplemented with 5 mM L-glycerophosphate, 100 nM dexamethasone, and 50 µg/mL ascorbic acid) for 28 days. Induction medium was changed every two days. Mineral deposits were identified by 0.1% Alizarin red S (pH 4.2, Sigma-Aldrich, St Louis, MO). Mineralized bone nodules were destained with 10% cetylpyridinium chloride and quantified by absorbance measurement at 405 nm with a spectrophotometer. Expression of osteogenic genes Bsp and Dmp1 was analyzed by real-time qPCR. All tests were performed in triplicate, at minimum.

### Quantitative real-time PCR

RNA was isolated from cells using RNAiso Plus (Takara, Tokyo, Japan) and reverse-transcribed to cDNA with the Revert Aid First Strand cDNA Synthesis Kit (Thermofisher Scientific, USA) according to the manufacturer's protocol. The real-time qPCR reaction was performed using a Quant Studio 6 Flex (Applied Biosystems, Foster City, CA, USA) with SYBR Premix Ex Taq (TaKaRa Biotechnology, Japan), and the relative expression levels were calculated with the comparative threshold cycle (∆∆CT) method as previously described 14, 15. All tests were performed in triplicate, at minimum. Primers are listed in the Appendix Table.

### Tissue sections and histological analysis

Consecutive tissue sections for histological analysis were prepared as previously described 14, 16, 17. Briefly, 5 μm tissue sections were prepared from each representative paraffin sample. After deparaffinization and rehydration, sections were stained with hematoxylin and eosin (H&E) (Solaibio, China) according to the manufacturer's recommended protocol. Immunofluorescence and immunohistochemical staining were also performed as previously described 14, 16, 17. Cells and tissue sections were examined under the fluorescence microscope (Olympus, IX73, Japan) and confocal microscope (Olympus FV1200, Japan), respectively. Primary and secondary antibodies, as well as the dilutions used in this study, are listed in the following paragraph. Negative control reactions were performed by substituting a normal IgG for the primary antibody.

Antibodies used included: rabbit anti-Sox2 (1:200, ab97959, Abcam, MA, USA); rabbit anti-Nanog (1:200, sc-33760, Santa Cruz, CA, USA); rabbit anti- POU5F1 (1:200, D121072, Sangon Biotech, Shanghai, China); rabbit anti-Ki67 (1:200, ab15580, Abcam, MA, USA); goat anti-DMP1 (1:200, sc-6551, Santa Cruz, CA, USA); rabbit anti-BSP (1:200, D261488, Sangon Biotech, Shanghai, China); rabbit anti-OCN (1:200, AP2002a 614487 , Zenbio, Chengdu, China); rabbit anti-periostin (1:200, sc-67233, Santa Cruz, CA, USA); rabbit anti-DSP (1:200, sc-33587, Santa Cruz, CA, USA); rabbit anti-HIF-1a (1:200, #36169 Cell Signaling Techology, MA, USA); mouse anti-actin (1:1000, Abcam, MA, USA) Alexa FluoR 488 goat anti-mouse (1:500, A11001, Invitrogen, Eugene, OR); Alexa FluoR 488 goat anti-rabbit (1:500, A11008, Invitrogen, Eugene, OR); Alexa FluoR 488 goat anti-mouse (1:500, A11001, Invitrogen, Eugene, OR); Alexa FluoR 555 goat anti-rabbit (1:500, A11008, Invitrogen, Eugene, OR); donkey anti-goat IgG-HRP (1:500, sc-2020, Santa Cruz, CA, USA); goat anti-rabbit IgG-HRP (1:500, ZB-2301, ZSGB-BIO, Beijing, China); goat anti-mouse IgG-HRP (1:500, ZB-2305, ZSGB-BIO, Beijing, China).

### Renal capsule transplantation and subcutaneous transplantation

8-week-old female Sprague-Dawley rats (Chengdu Dashuo Experimental Animal Co. Ltd, Chengdu, China) were used in transplantation experiments for evaluating the function of cementogenesis differentiation and inductive ability to form dentin *in vivo*.

Renal capsule transplantation can be used to evaluate both the function of cementogenesis differentiation and inductive ability to form dentin. Approximately 2.0 × 10^6^ cells (2D monolayer HERS cells, HERS spheroids, DPC alone, or 2D monolayer HERS cells/HERS spheroids mixed with DPCs in a 1:2 ratio) were mixed with 40 mg hydroxyapatite/tricalcium phosphate (HA/TCP) ceramic powder (Engineering Research Center in Biomaterials, Sichuan University) and transplanted into the renal capsule.

Subcutaneous transplantation was used to assess the inductive ability to form dentin of HERS cells cultured using different methods. Seed cells (DPC alone or 2D monolayer HERS cells/ HERS spheroids mixed with DPCs in a 1:2 ratio) were resuspended with Matrigel, at a concentration of approximately 1.0 × 10^8^ cell/ml. Then, 20 μl of this mixed Matrigel was injected into the center of the treated dentin matrix (TDM) and transplanted subcutaneously into SD rats after overnight culture in the medium.

Grafts were obtained 8 weeks post-operation, fixed with 4% paraformaldehyde, decalcified with 10% EDTA for 6 weeks, and then embedded in paraffin for further analysis.

### RNA-Seq analysis

RNA was extracted with RNAiso Plus (Takara, Tokyo, Japan) according to the manufacturer's protocol. Sequencing was performed on the Illumina platform. The raw RNA-seq reads were aligned to the *Rattus norvegicus* genome (rn6) by hisat2 (version:2.1.0). Mapped reads were counted by featureCounts (version: 1.6.2) and gene expression was calculated by R and the DESeq2 package 18. All analysis was performed in R using different packages. Correlation heatmap and principal component analysis (PCA) was performed with DESeq2 based on the gene expression data. Significantly differentially expressed genes (DEGs) (logFoldChange ≥1, p-adjusted < 0.05) between HERS spheroids and 2D monolayer HERS cells were assessed using DESeq2. Additionally, Gene Ontology (GO) and Kyoto Encyclopedia of Genes and Genomes (KEGG) enrichment analyses of DEGs were performed using the clusterProfiler package 19.

### Statistical analysis

Statistical analysis was performed with SPSS 20.0 software. All data were expressed as mean ± standard deviation (SD). Statistical significance was assessed by using the Student's t-test for two groups and one-way ANOVA for more than 2 groups. P < 0.05 was considered to be statistically significant.

## Results

### HERS spheroids were superior in stem cell characteristics compared to 2D monolayer HERS cells

**Figure 1:**
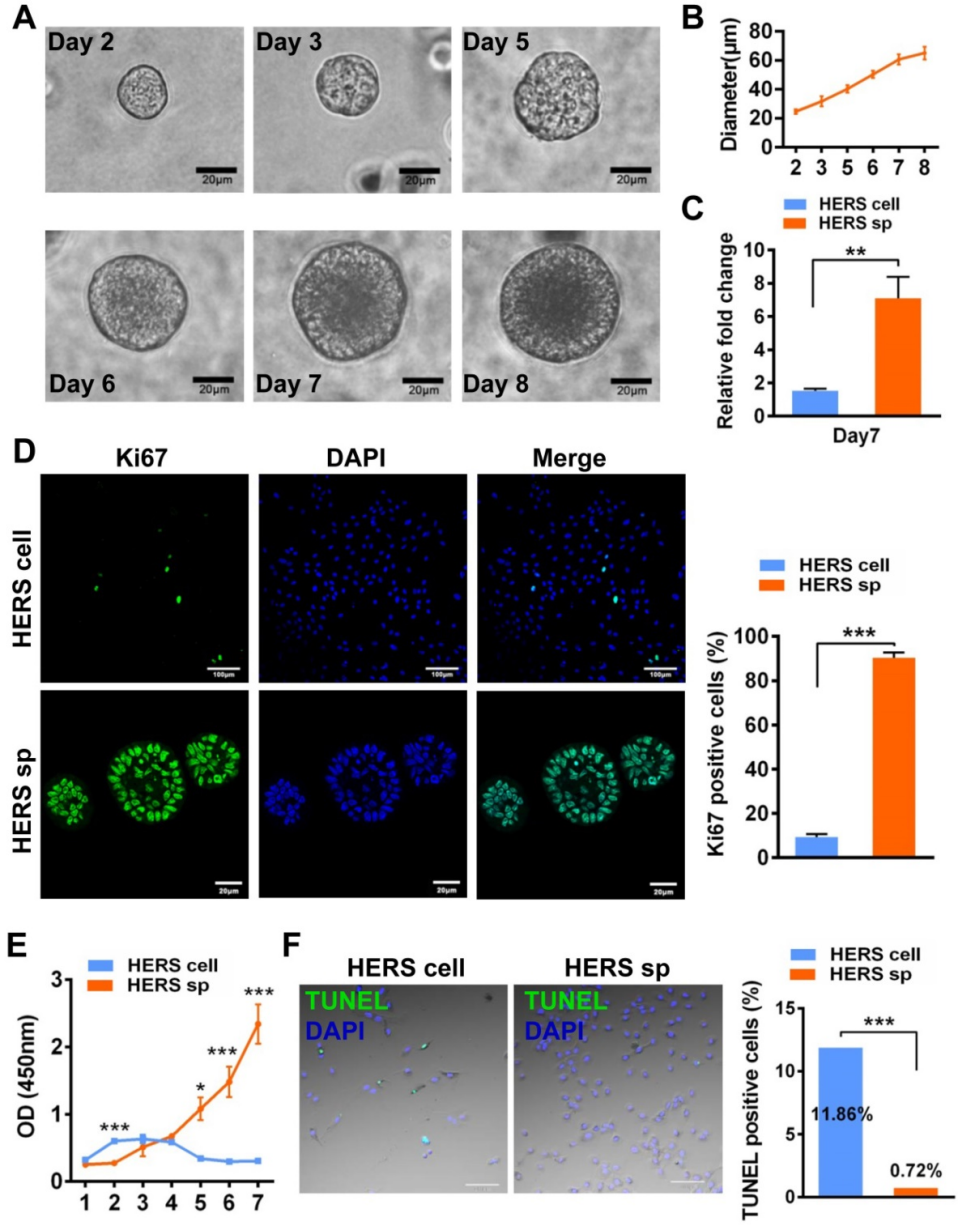
** HERS spheroids expanded steadily and contributed to cell proliferation.** (A) Time course representative images of HERS spheroid growth showing HERS spheroid formation progress. (B) Change in spheroid diameter was recorded daily, revealing that HERS spheroids steadily increase and slow down at day 7. (C) Cells were counted to compare the expansion efficiency and the relative fold change to the initially seeded cells after 7 days of culture; the higher expansion efficiency of HERS spheroids is clear. (D) Ki67 was detected by immunofluorescence in most of the HERS spheroids but in few of the 2D monolayer HERS cells, supporting findings that the HERS spheroids had higher proliferation ability. (E) Growth curves were created based on CCK-8 assay and showed that cells from HERS spheroids had higher proliferation capacity than 2D monolayer HERS cells after the first day (n=5). (F) There were far more TUNEL-positive cells in the 2D monolayer HERS cells than in cells digested from HERS spheroids. Scale bars are shown, *** P < 0.001; ** P< 0.01; * P < 0.05.

Primary HERS cells at passage 1 were cultured with different methods: one with HERS spheroids formation methods (HSCM), and another with traditional 2D monolayer methods. Both the HERS spheroids and 2D monolayer HERS cells expressed the epithelial and mesenchymal cell markers of primary cells, indicating that all cells maintained the characteristics of both epithelial and mesenchymal cells 14 (Figure S1A-C). Within 8 days, among cells cultured with HSCM, HERS cells gradually expanded and grew into spheroids about 70 μm in size (Figure 1A-B). Cell counts were used to compare expansion efficiency. After 7 days of culture, we found HERS spheroids had higher cell numbers than 2D monolayer HERS cells and exhibited significantly higher fold-change compared to the initial number of seeded cells (Figure 1C). At the same time, Ki67, the classical marker of proliferation, can be detected in almost all nuclei in HERS spheroids, but only in a few nuclei of 2D monolayer HERS cells (Figure 1D). To further compare their proliferative capacity under the same conditions, HERS spheroids were digested into single cells and the CCK8 assay was applied. CCK8 showed that the proliferation of 2D monolayer HERS cells stagnated and even diminished from day 2, while cells from HERS spheroids kept steadily expanding (Figure 1E). Furthermore, we detected cells at day 6 with the TUNEL assay and found that there were more clearly FITC-labeled TUNEL-positive cells in the 2D monolayer HERS cells than in cells digested from HERS spheroids, indicating that 2D monolayer HERS cells may undergo apoptosis, meaning that HERS spheroids are a better way to expand HERS cells (Figure 1F).

**Figure 2:**
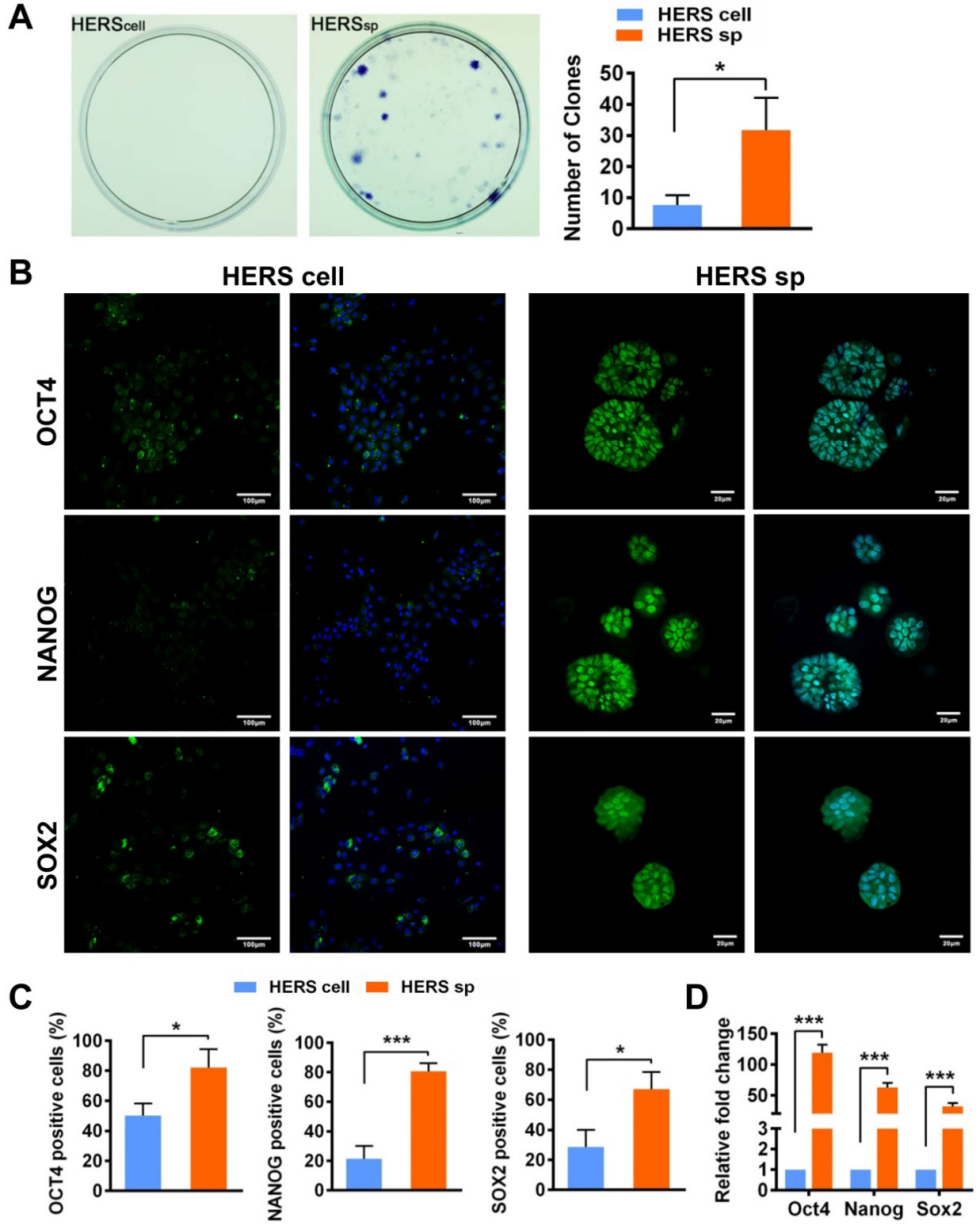
** HERS spheroids enhanced the stem cell traits of HERS cells.** (A) The CFU assay was conducted to compare the colony formation ratio between the 2D monolayer HERS cells and HERS spheroids. More cell colonies (purple dots) can be observed in the HERS spheroids groups. (B) Immunofluorescence staining images showed that both the ratio and intensity of Nanog-, Sox2-, and Oct4 (green)-positive cells in HERS spheroids were higher than in 2D monolayer HERS cells. Nuclei are stained with DAPI (blue). (C) The proportion of Nanog-, Sox2-, and Oct4-positive cells in HERS spheroids and 2D monolayer HERS cell groups were quantified. (D) RT-qPCR analysis of the relative expression of Nanog, Sox2, and Oct4. Scale bars are shown, *** P < 0.001; ** P< 0.01; * P < 0.05.

Self-renewal is another key characteristic of stem cells. The CFU assay demonstrated that cells from HERS spheroids generated more clones than the 2D monolayer HERS cells, revealing that cells from HERS spheroids maintained better self-renewal capacity (Figure 2A). Moreover, immunofluorescence results indicated that most cells in the HERS spheroids were positive for Nanog, Sox2, and Oct4 (Figure 2B-C), widely accepted markers of multipotency and self-renewal for cells 20, 21. RT-qPCR also showed that the expression of Nanog, Sox2, and Oct4 in HERS spheroid was significantly higher than that in 2D monolayer HERS cells (Figure 2D). Taken together, these results proved that the HERS spheroids had better proliferation ability and self-renewal capacity, and maintained better stem cells traits than did 2D monolayer HERS cells.

### HERS spheroids maintained the functions of HERS better *in vitro* and *in vivo*

**Figure 3:**
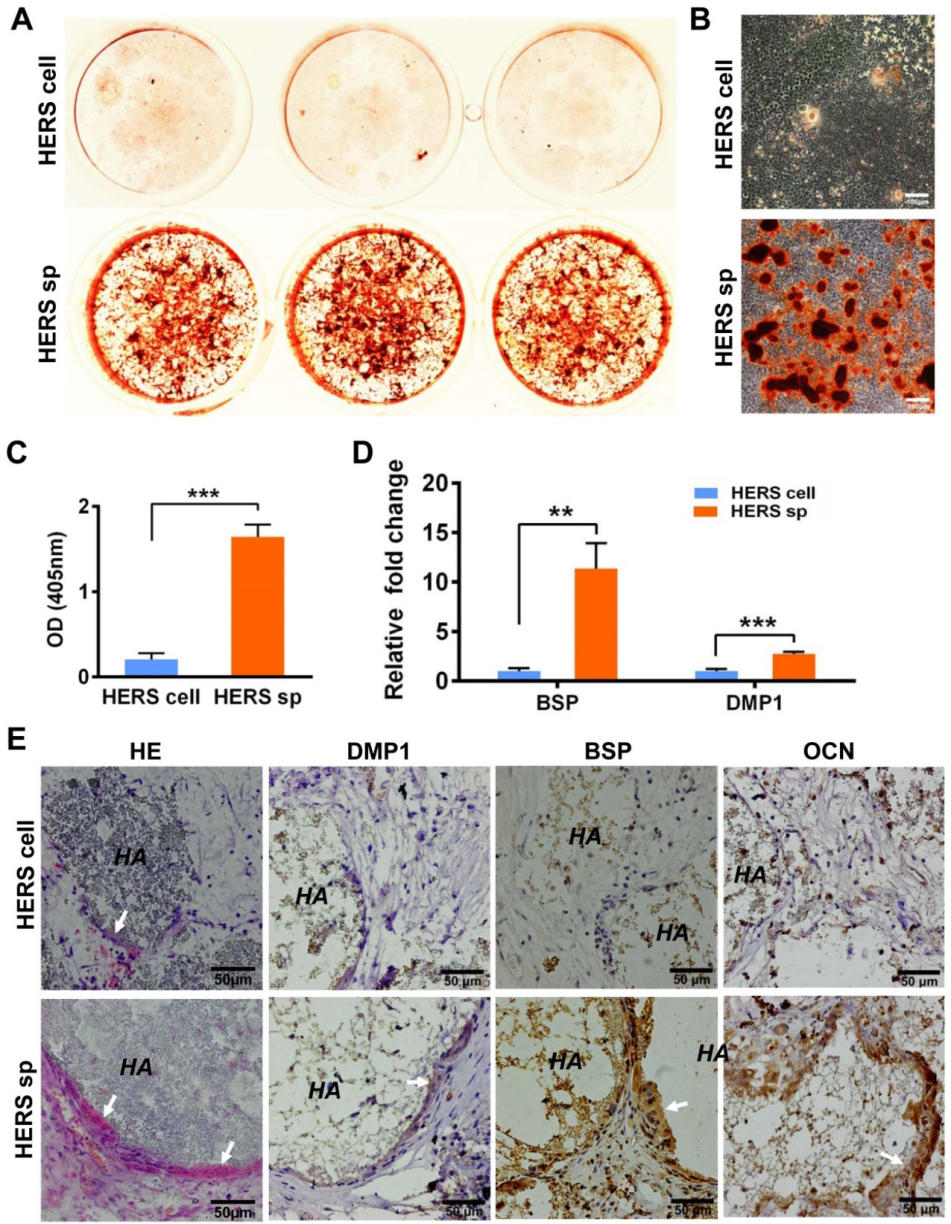
** HERS spheroids, rather than 2D monolayer HERS cells, maintained the cementogenesis potential both *in vitro* and *in vivo*.** (A) The alizarin red staining of mineralized deposits formed by 2D monolayer HERS cells (upper) and HERS spheroids (lower) indicated that more mineralized deposits formed in the HERS spheroids groups after induction. (B) Light microscope images of alizarin red staining also revealed that more mineralized deposits formed in the HERS spheroids groups, also. (C) Quantification of the alizarin red staining showed a consistent trend. (D) RT-qPCR analysis of the relative expression of cementogenesis-associated genes BSP and DMP1 after induction. Their relative expression level is higher in the HERS spheroids groups. (E) Cementogenesis potential was compared *in vivo*. H&E staining revealed a thin layer of cementum-like tissue generated on the surface of HA/TCP particles in the HERS spheroids groups, but not in the 2D monolayer HERS cells transplant (n=4). IHC showed the cementum-like tissues were positive for DMP1, BSP, and OCN antibody compared to the nearly-negative stain in the 2D monolayer HERS cells transplants. Scale bars are shown, *** P < 0.001; ** P< 0.01; * P < 0.05.

To clarify whether HERS spheroids still possessed the key function of HERS, we further assessed the differentiation potential and inductive ability to form dentin both* in vitro and in vivo*. After mineralization induction *in vitro*, a bright-red field was observed after Alizarin Red S staining was performed in the HERS spheroids groups (Figure 3A), and relatively more Alizarin Red-positive mineralized nodules can be observed in the HERS spheroids groups (Figure 3B). The quantitative result was also consistent (Figure 3C). Furthermore, RT-qPCR revealed that osteogenic-associated genes, including bone sialoprotein (BSP) and dentin matrix protein 1 (DMP1), were highly activated in the HERS spheroids groups (Figure 3D) compared to the 2D monolayer HERS cells groups. These results demonstrated the enhanced mineralization potential of HERS spheroids *in vitro*.

To confirm the results *in vivo*, 2D monolayer HERS cells or HERS spheroids mixed with Matrigel and HA/TCP particles were transplanted into the renal capsule of SD rats. After eight weeks, H&E staining showed thin layers of mineralized structure at the surface of HA/TCP particles in the HERS spheroids transplants, while little mineralization was observed in the HERS cell grafts (Figure 3E). Moreover, positive signals for BSP, DMP1, and OCN were clearly detected in the HERS spheroids group, but signals were negative or faint in HERS cell transplants, as showed by immunohistochemical staining (Figure 3E). The mineralized structures were HERS-originated, indicating that HERS spheroids, not 2D monolayer HERS cells, maintained their cementogenesis differentiation potential after expansion.

**Figure 4:**
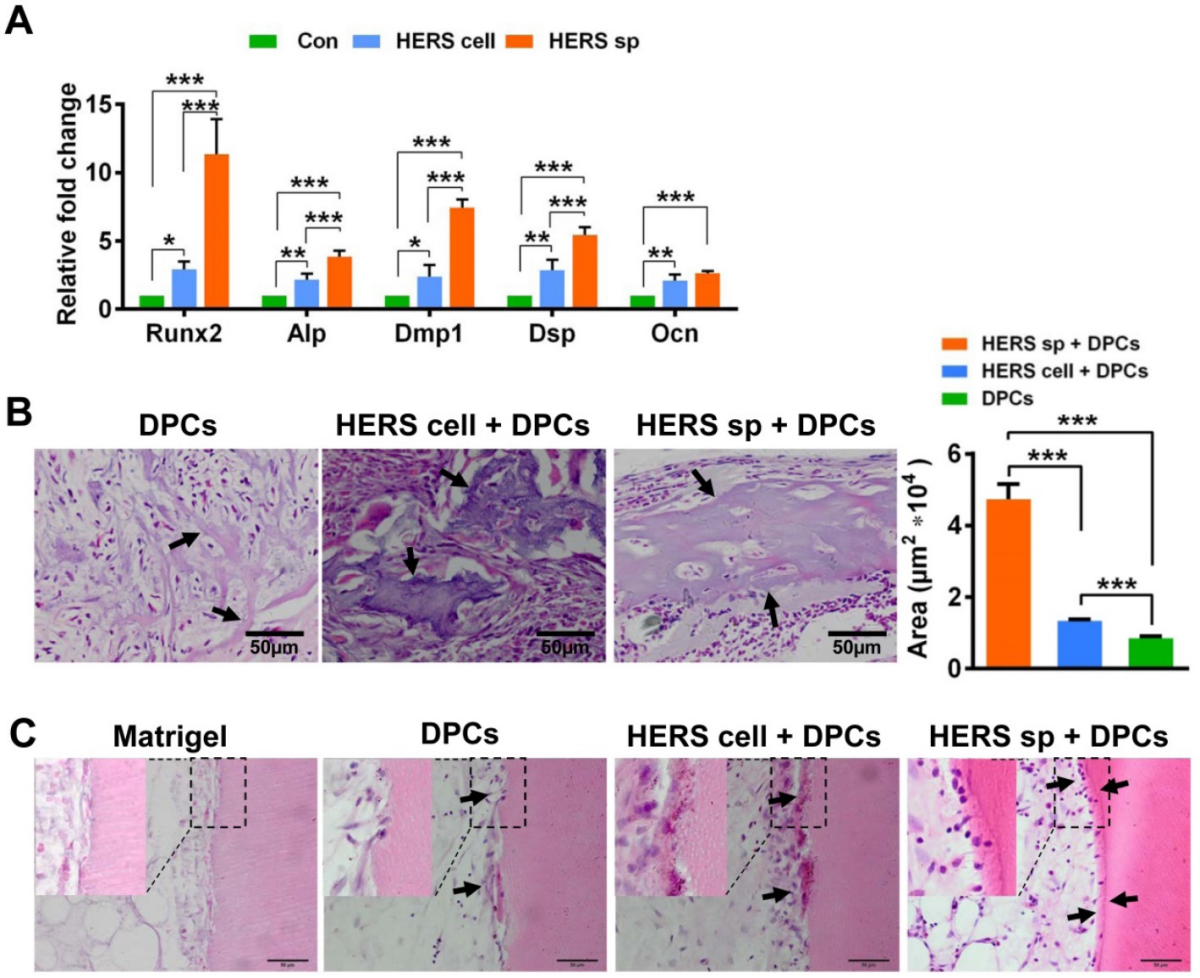
** HERS spheroids have better inductive capacity both *in vitro* and *in vivo* compared to 2D monolayer HERS cells.** (A) RT-qPCR analysis of the relative expression of Runx2, Alp, Dmp1, Dsp, and Ocn. These were elevated in both HERS spheroids and 2D monolayer HERS cell groups compared to control groups, but the level of up-regulation in HERS spheroids was greater. (B) *In vivo* osteogenesis inductive potential was compared with H&E (n=4) staining, which showed that the osteodentin-like tissues mainly appeared HERS spheroids and 2D monolayer HERS cells groups, especially in the HERS spheroids groups. Quantification of the hard tissue showed the same trend. (C) Dentin and dental pulp regeneration were compared with H&E staining. Clear and regular new dentin-like tissues and odontoblast-like cells can be observed in the HERS spheroids groups; some disordered dentin-like tissues and odontoblast-like cells appeared in the 2D monolayer HERS cells; few disordered dentin-like tissues and odontoblast-like cells formed in the DPCs groups; and only adipose tissues could be found in the Matrigel groups (n=3). Scale bars are shown, *** P < 0.001; ** P< 0.01; * P < 0.05.

To test induced differentiation of DPCs and dentin formation ability, conditional medium from HERS spheroids or 2D monolayer HERS cells was collected to induce DPCs *in vitro*. After induction lasting 7 days, the expression of odontoblastic-associated genes Runx2, Alp, Dmp1, Dsp, and Ocn was activated in DPCs treated with medium from both HERS spheroids and 2D monolayer HERS cells (Figure 4A), indicating that both cell groups maintained induction capacity. However, the level of most of these up-regulated genes was higher in the spheroid cells than in the 2D monolayer cells (Figure 4A). This difference indicates that the HERS spheroids may have better induction capacity than HERS cells grown in a 2D monolayer.

To further verify this phenomenon *in vivo*, 2D monolayer HERS cells/HERS spheroids mixed with DPCs, or DPCs alone, were transplanted into the renal capsule of adult SD rats. Eight weeks after transplantation, mineralized tissues were generated in all groups, as shown by H&E staining (Figure 4B). Relative order and clearly mineralized tissues can be observed in the DPCs combined with HERS spheroids groups; medium-sized but still-disordered mineralized tissues appeared in the DPCs mixed with 2D monolayer HERS cells; while in the DPCs-only group, only a few tiny and disordered mineralized tissues formed (Figure 4B). This result is consistent with our *in vitro* study. Together, both the 2D monolayer HERS cells and HERS spheroids demonstrated induction capacity, but HERS spheroids were superior in this regard.

Furthermore, DPCs mixed with HERS spheroids or 2D monolayer HERS cells were transplanted into the TDM, with Matrigel and DPCs used as controls. After 8 weeks, a layer of ordered mineralized tissues was found to be located in the surface of TDM, with cells clearly aligned on one side of the ordered mineralized tissues. Some disordered mineralized tissue was apparent in the DPCs mixed with 2D monolayer HERS cells groups. However, in the control group, few disordered mineralized tissues were observed in the DPCs groups, and only adipose tissue appeared in the Matrigel groups (Figure 4C). These results demonstrated the induction ability of both 2D monolayer HERS cells and HERS spheroids, even without HA/TCP.

Taken together, these findings indicate that HERS spheroids showed obvious advantages in cell characteristics and maintained the key functions of HERS tissues.

### HERS spheroids formation and expansion rely on the HIF-1 pathway

In view of distinct cell characteristics, we compared the transcriptome difference between HERS spheroids and 2D monolayer HERS cells by bulk RNA sequence to further understand the mechanism. HERS primary cells at passage 1 were included as a control.

**Figure 5:**
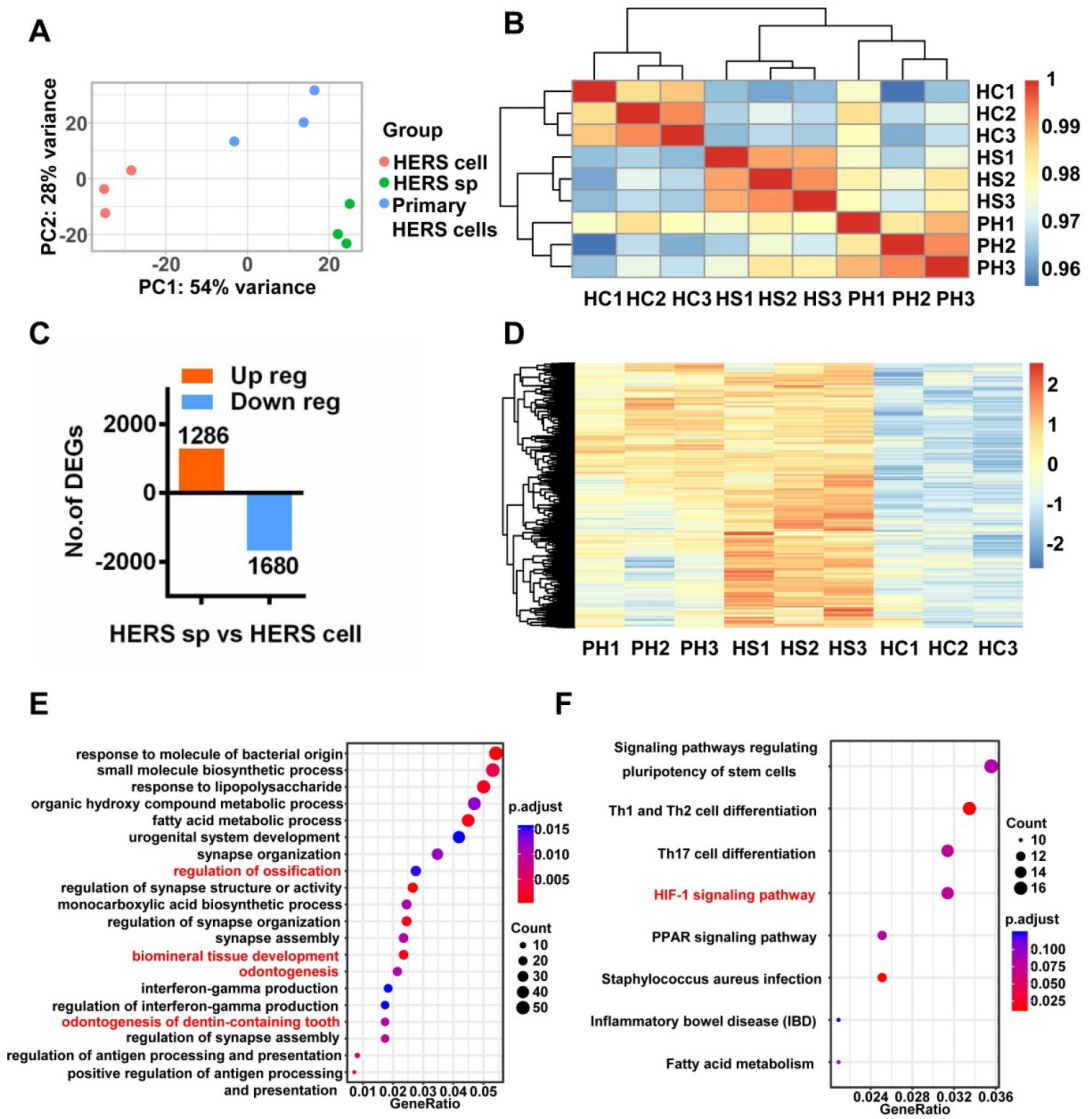
** RNA-seq was applied to investigate mechanisms.** (A) Principal component analysis (PCA) revealed the variance among each group and indicated that HERS spheroids were similar to HERS primary cells. (B) The correlation heatmap results were consistent with PCA and showed that there was better homogeneity within the HERS spheroids groups. (C) Among the 2966 differentially expressed genes (DEGs) between HERS spheroids and 2D monolayer HERS cells, 1286 genes were up-regulated and 1680 genes were down-regulated. (D) Heatmap presenting the up-regulated DEGs between HERS spheroids and 2D monolayer HERS cells. (E) Gene ontology (GO) analysis of the up-regulated genes revealed 4 of the top 20 enriched biological processes were odontogenesis-related, consistent with results indicating that HERS spheroids maintained cementegenesis potential. (F) KEGG enrichment analysis presented several pathways highly related to stem cell traits. HC: 2D monolayer HERS cells; HS: HERS spheroids; PH: Primary HERS cells.

**Figure 6:**
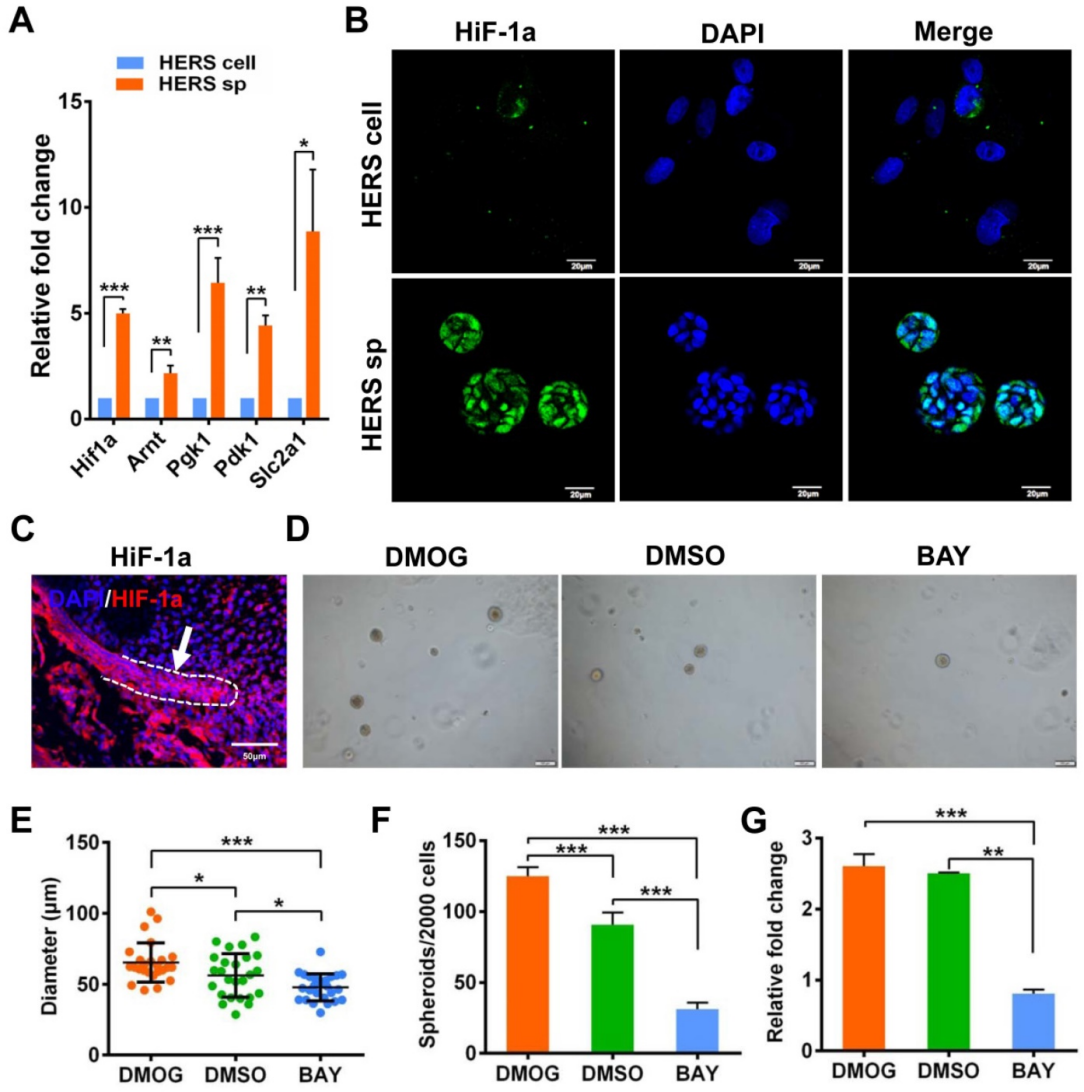
** Generation and expansion of HERS spheroids rely on the HIF-1 pathway.** (A) RT-qPCR analysis of expression of representative genes in the HIF1 pathway. The expression of Hif1a, Arnt, Pgk1, Pdk1, and Slc2a1 was relatively higher in the HERS spheroids groups, consistent with the results of RNA-seq analysis. (B) Immunofluorescence staining was used to verify the expression of HIF-1a protein. The HIF-1a (green) signal was located in many of the nuclei of cells in HERS spheroids, but in only a few nuclei in the 2D monolayer HERS cells. (C) HIF-1a expression can be detected in HERS tissues. (D) DMOG (a HIF-1 activator) and BAY (a HIF-1 inhibitor) were applied to perform gain- and loss-of-function assays. The sphere-formation rate (E) and the diameter of spheroids (F) were severely inhibited by HIF-1 inhibitor and enhanced by HIF-1 activator, indicating that the HIF-1 pathway is important for the formation and expansion of HERS spheroids. In addition, the expansion of 2D monolayer HERS cells was also inhibited by BAY (G). *** P < 0.001; ** P < 0.01; * P < 0.05.

Interestingly, Principal Component Analysis (PCA) (Figure 5A) indicated that different gene expression profiles existed among HERS primary cells, HERS spheroids, and 2D monolayer HERS cells. However, the variation between 2D monolayer HERS cells and HERS primary cells was more obvious than between HERS spheroids and HERS primary cells (Figure 5A). This finding was also supported by correlation analysis among these three groups (Figure 5B), indicating a more-similar gene expression profile between HERS spheroids and primary cells. We further focused on differentially expressed genes (DEGs) between HERS spheroids and 2D monolayer HERS cells. As a result, we found 2966 DEGs, with 1286 up-regulated and 1680 down-regulated (Figure 5C-D). According to GO analysis, up-regulated genes in HERS spheroid were enriched in biological processes related to regulation of ossification, biomineral tissue development, odontogenesis, and odontogenesis of dentin-containing tooth. This result revealed the activation of odontogenesis-related processes in HERS spheroids (Figure 5E), which may explain why HERS spheroids have mineralization potential, while 2D monolayer HERS cells do not. Moreover, according to the KEGG enrichment analysis, several pathways highly related to the stem-cell traits were activated in HERS spheroids, including pathways regulating pluripotency of stem cells, HIF-1 signaling pathway, PPAR signaling pathways, and fatty acid metabolism pathway(Figure 5F). Down-regulated genes were related to functions such as cell migration, adhesion, and motility (Figure S2A-C). Because the HIF-1 pathway plays an important role in cell biology processes including stemness maintenance, cell proliferation, survival, and glucose metabolism 22, we further focused on this pathway and verified the activation of HIF-1 pathway at the mRNA and protein levels using RT-qPCR (Figure 6A) and immunofluorescence staining (Figure 6B) in HERS spheroids. These results revealed that the HIF-1 pathway was activated in HERS spheroids. In addition, the HIF1a signal can be detected in HERS tissues (Figure 6C), indicating that activation of the HIF-1 pathway may be important for HERS cells. In order to verify the function of the HIF-1 pathway, Dimethyloxallyl Glycine (DMOG, a HIF-1 activator) and BAY-87-2243 (BAY, a HIF-1 inhibitor) were applied for gain- and loss-of-function assays. After culture with HSCM for 7 days, compared to the control groups, the formation rate and size of HERS spheroids decreased in the BAY groups and increased in the DMOG groups (Figure 6D-F), suggesting the importance of the HIF-1 pathway in HERS spheroid formation and expansion. In addition, the expansion of 2D monolayer HERS cells was also inhibited by BAY (Figure 6G).

Together, these data demonstrate that the HIF-1 pathway is important for the formation and expansion of HERS spheroids that contribute to the stem cell traits and self-renewal property of HERS cells. In addition, the activation of odontogenesis-related process in HERS spheroids may play an important role in preserving cemetogenesis functions.

## Discussion

Mesenchyme and epithelium are two necessary components in tooth development. HERS is the indispensable epithelium that participates in tooth root formation, and it is a potential cell source for tooth root-related tissue engineering and regeneration once the limitation on cell expansion can be overcome.

A previous study constructed immortalized HERS cells for cementum and dentin regeneration 14. These immortalized cells were more suitable in terms of cell characteristics and mechanical research than direct tissue regeneration due to the potential risk of tumorigenicity. In this study, we tried a 3D culture method that may provide enhanced cell-cell interactions and can mimic the natural microenvironment of the tissue *in vivo* 12. By allowing cells to maintain their innate morphology, we observed significant differences in cellular phenotype and biological function between cells cultured in monolayer and 3D environments 10. 3D culture also contributes to the enriched expression of stemness-associated genes, enhancing multipotent differentiation capacity 11, 13. In order to select appropriate methods for HERS cells, we tried several representative 3D culture methods and finally established HSCM, in which a single HERS cell can gradually form a spheroid that mimics a typical 3D clone-like growth. Impressively, the stem cell characteristics of cells in HERS spheroids, such as cell proliferation potential, self-renewal, and differentiation ability, showed obvious advantages relative to, and were distinct from, the 2D monolayer HERS cells. Moreover, the HERS spheroids maintained the key function of primary HERS cells both *in vitro* and *in vivo*, indicating that HSCM may be an ideal system for HERS cells production. It is worth mentioning that previous studies also showed that 3D culture enhances expression of Nanog, Oct4, and Sox2, as well as boosts reprogramming through an accelerated mesenchymal-to-epithelial transition and increased epigenetic remodeling 23-26.

In order to uncover the mechanism underlying their distinct cell characteristics, RNA-seq was applied to spheroid cells, and several pathways highly related to stem cells were revealed. Among them, HIF-1 pathway was one of the top pathways in the enrichment ranking that got our attention. Previous work revealed that HIF1a was a key regulator of the cell and body during hypoxia 27-31. Additionally, HIF-1 pathway activation seems to be a common characteristic in various stem cells and is indispensable for maintaining their stemness in their “hypoxic niche” 27-31. Moreover, HIF1a can be observed in primary HERS cells and is maintained in the HERS spheroids rather than in the 2D monolayer HERS cells, at both the RNA and protein levels (Figure 6A-B). Furthermore, the survival of HERS cells and HERS spheroid formation were severely inhibited by HIF1a inhibitor, suggesting that HIF1a is essential for the survival and proliferation of HERS cells. When cultured with HSGM, the solid surrounding microenvironments and spheroid-like growth pattern provided an appropriate oxygen concentration for HERS cells, contributing to the advantages of the HERS spheroid use.

HIF1a has also been reported to mediate cell metabolism. For example, HIF1a leads to the upregulation of PDK1, which accelerates glycolysis and plays a critical role in promoting stem-cell traits of breast cancer stem cells 22. However, according to our experimental results, the fatty-acid-related metabolism pathway, rather than glycolysis, was activated in HERS spheroids (Figure 5F). Although fatty acid metabolism is related to stem cells 32, 33, the relationship between the HIF-1 pathway and fatty acid metabolism is still unclear and needs to be further explored.

With regard to the key functions of HERS cells, our study showed that HERS spheroids rather than 2D monolayer HERS cells maintain mineralization ability both *in vitro* and *in vivo* (Figure 3A-E), consistent with the activation of odontogenic-related biological processes in the HERS spheroids, according to RNA-seq results (Figure 5E). In terms of the inductive ability to form dentin, more and better osteodentin was generated when DPCs were transplanted with HERS spheroids than with 2D monolayer HERS cells, indicating that HERS spheroids had better induction ability. More obvious dentin-like tissues were generated in the HERS spheroids groups in the subcutaneous transplantation model, as well. These results indicate that HERS spheroids maintain the functions of HERS cells better than HERS cells grown in 2D monolayers.

There are still some controversial conclusions regarding the mineralization potential of HERS cells. Indeed, some research has found that Amel 34, Runx2 35, vimentin 35, or ALP 36-38 were undetectable in the Hertwig's epithelial root sheath, and that HERS disintegrates prior to cementum formation 34. This indicates that there may be no EMT or cementogensis promoted by HERS cells. However, several issues should be noted. First, Amel is the marker of ameloblast, which is located in the tooth crown and participates in enamel formation, while HERS is located in the tooth root region and does not take part in the enamel-formation process. It is therefore easy to understand that Amel expression is undetectable in HERS. Next, according to our previous study, vimentin, a mesenchymal cell marker, can be detected in HERS tissues, and its expression increases gradually 8. Additionally, some research has shown that BSP 7, collagen 7, vimentin 8, AMBN 39, OCN 39, and ALP 39 can be detected in HERS, indicating that they may take part in cementum formation. In the present study, we confirmed that HERS cells differentiation or induction DPCs formed the mineralized tissues both *in vivo* and *in vitro*.

## Conclusion

In summary, we constructed a 3D culture method, HSCM, which can generate HERS spheroids. HERS spheroids not only significantly promote the expanding efficiency of HERS cells, but also optimize the cell characteristics and functions of HERS cells. Additionally, we found that the HIF-1 pathway is important to HERS spheroid formation and survival. Moreover, HERS spheroids may usher in a new era in the field of tooth-related tissue regeneration, as they can be a cell source supporting regeneration and further mechanistic research.

## Supplementary Material

Supplementary figures.

## Abbreviations

2D: two dimensions
3D: three dimensions
CCK8: cell Counting Kit-8
CFU: colony-forming unit
DPCs: dental papilla cells
HERS: Hertwig's epithelial root sheath
EMT: epithelial-mesenchymal transitions
HIF-1: hypoxia inducible factor-1**
HSCM: HERS spheroids culture methods
MC: methylcellulose
TDM: treated dentin matrix
