# Supplementary material for: Therapeutic potential of HERS spheroids in tooth regeneration
Source: Theranostics. 2020 Jun 12;10(16):7409–21. doi: 10.7150/thno.44782 (PMC7330840; doi:10.7150/thno.44782)
Supplement: Supplementary file 1 — Supplementary figures. [file thnov10p7409s1.pdf]

Supplementary information

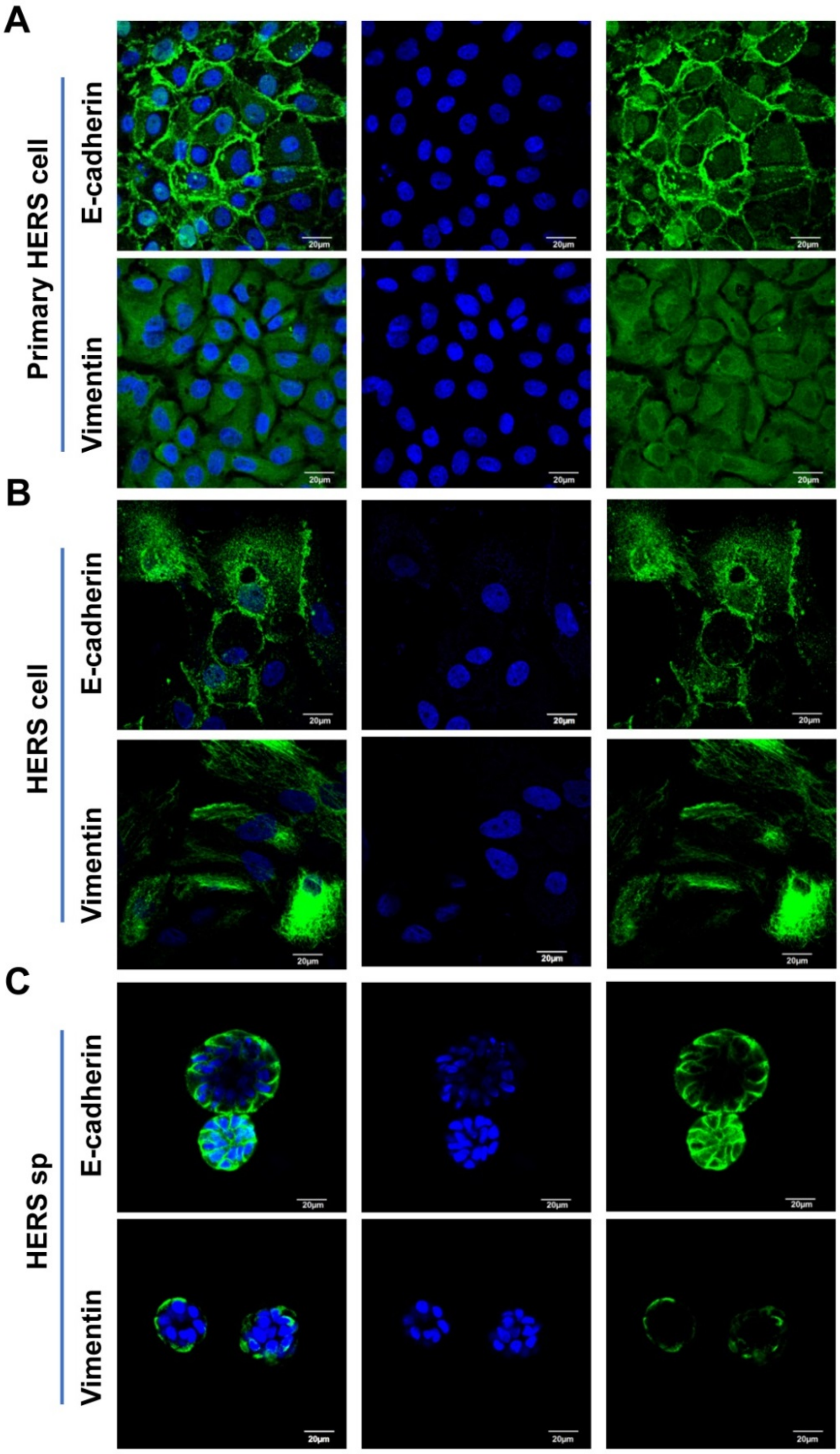

Figure S1: Detecting the expression of E-cadherin and vimentin among primary HERS cells, 2D monolayer HERS cells, and HERS spheroids. Primary HERS cells (A), 2D monolayer HERS cells (B), and HERS spheroids (C) all expressed the epithelial cell marker E-cadherin and the mesenchymal cell marker vimentin.

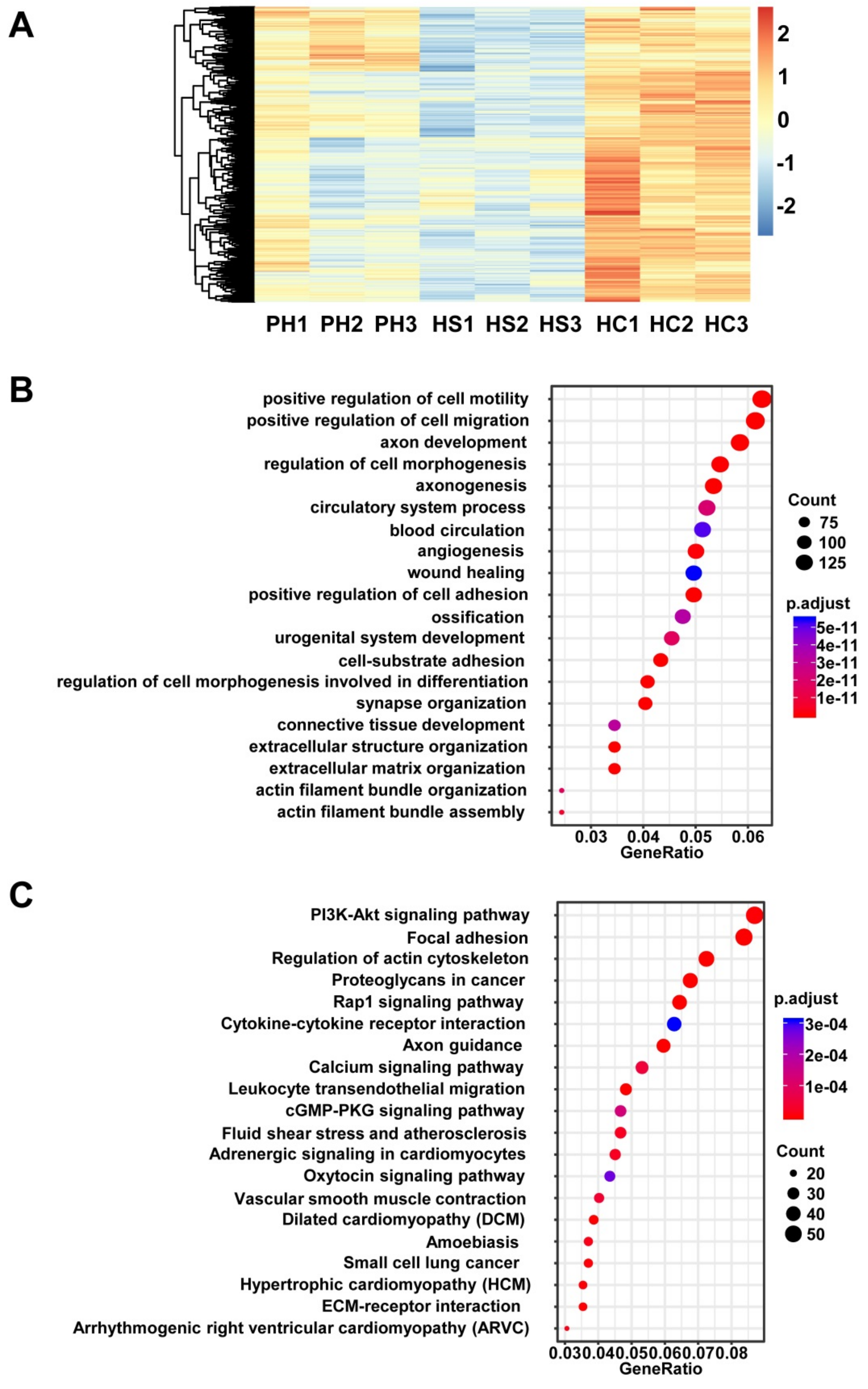

Figure S2: Analysis of the down-regulated genes in HERS spheroids compared with 2D monolayer HERS cells. (A) The down-regulated genes between HERS spheroids and 2D monolayer HERS cells are presented with Heatmap. (B) Gene ontology (GO) analysis of down-regulated genes (top 20). (C) KEGG enrichment analysis of the down-regulated genes (top 20). HC: 2D monolayer HERS cells; HS: HERS spheroids; PH: Primary HERS cells.
